# Association of remnant cholesterol and cholesterol, high-density lipoprotein, and glucose index with unfavorable outcomes after intravenous thrombolysis in acute ischemic stroke: a dual-center cohort study

**DOI:** 10.3389/fimmu.2026.1797414

**Published:** 2026-03-31

**Authors:** Zhuang Zhu, Hualin Wang, Yao Geng, Bo Du, Kefan Yi, Zefu Yin, Yihang Xu, Min Xu, Jiaxin Shi, Yongsheng Yuan, Deqin Geng, Kezhong Zhang

**Affiliations:** 1 Department of Neurology, The First Affiliated Hospital with Nanjing Medical University, Nanjing, Jiangsu, China; 2 The First School of Clinical Medicine, Nanjing Medical University, Nanjing, Jiangsu, China; 3 Department of Neurology, The Affiliated Hospital of Xuzhou Medical University, Xuzhou, Jiangsu, China; 4 Clinical Nutrition Department, Shanghai Deji Hospital, Qingdao University, Shanghai, China

**Keywords:** acute ischemic stroke, cholesterol, high-density lipoprotein, and glucose index, intravenous thrombolysis, prognosis, remnant cholesterol

## Abstract

**Background and purpose:**

This study aimed to investigate the independent and joint effects of two novel lipid-derived biomarkers—Remnant cholesterol (RC) and Cholesterol, high-density lipoprotein, and glucose (CHG) index—with hemorrhagic transformation (HT) and poor prognosis in patients with acute ischemic stroke (AIS) after intravenous thrombolysis (IVT).

**Methods:**

In this dual-center retrospective cohort study, 4403 AIS patients undergoing IVT were analyzed. RC and the CHG index were calculated from routine baseline biochemical measurements. The primary outcome was HT, and the secondary outcome was poor prognosis at 90 days (modified Rankin Scale [mRS] score > 2). Multivariable logistic regression and restricted cubic spline (RCS) models were used to assess linear and non-linear associations. Subgroup and receiver operating characteristic (ROC) analyses were conducted to evaluate robustness and discriminative ability. Mediation analysis explored the potential mediating role of the systemic immune-inflammation index (SII).

**Results:**

Patients with HT or a poor prognosis had lower RC and CHG levels. Multivariate logistic regression analysis revealed that both RC and CHG were negatively associated with the risk of HT and with a poor prognosis. The joint effects showed that patients with higher levels of RC and CHG had a significantly lower risk of HT (OR = 0.18, 95% CI: 0.12–0.25) and poor prognosis (OR = 0.40, 95% CI: 0.32–0.49). Subgroup analyses confirmed the consistency of these negative associations across age, sex, baseline NIHSS, and vascular comorbidities. The RCS models showed a non-linear association between RC and poor prognosis (*P* for non-linearity < 0.05) but a linear trend with HT (*P* > 0.05). In contrast, CHG showed a U-shaped non-linear association with both outcomes (*P* < 0.05). Moreover, the combination of RC and CHG had better predictive efficacy for HT (AUC = 0.750, 95% CI: 0.727–0.773, *P* < 0.001) and poor prognosis (AUC = 0.721, 95% CI: 0.705–0.737, *P* < 0.001) than alone. Mediation analysis further showed that SII partially mediated these associations.

**Conclusion:**

High RC and CHG levels were associated with a lower risk of HT and poor prognosis in AIS patients undergoing IVT. Integrating these biomarkers enhances predictive performance, supporting their potential utility in pre-thrombolysis risk stratification and personalized treatment decision-making.

## 1 Introduction

Stroke remains a significant public health challenge in China, with approximately 343 cases per 100,000 population reported annually—representing the highest incidence worldwide. Acute ischemic stroke (AIS) is the predominant subtype (1, 2). Intravenous recombinant tissue plasminogen activator (IV-rtPA) is the most effective evidence-based treatment for eligible AIS patients (3). However, despite its proven benefits, IV-rtPA is associated with a substantial risk of hemorrhagic transformation (HT), a complication that can exacerbate neurological injury, worsen functional outcomes, and increase mortality (4). Identifying modifiable risk factors and early predictive biomarkers is therefore essential for timely intervention and risk stratification.

Although lipid parameters are traditionally considered important vascular risk factors, recent studies have highlighted the so-called “lipid paradox,” in which lipid levels show paradoxical associations with outcomes in acute cardiovascular and cerebrovascular diseases (5). Evidence regarding the relationship between cholesterol levels and stroke risk remains inconsistent: some studies report a positive association, whereas others suggest neutral or even inverse association (6). These discrepancies indicate that single lipid markers—such as LDL-C or HDL-C—may be insufficient to capture the complexity of lipid metabolic disturbances during acute cerebrovascular events or to reflect their potential influence on inflammation, endothelial injury, and HT susceptibility.

Beyond lipid imbalance, a growing body of evidence suggests that the overall homeostasis of glucose–lipid metabolism plays a crucial neuroprotective role during acute cerebral ischemia. Adequate glucose availability is necessary to sustain energy metabolism under ischemic stress, while sufficient lipid reserves help maintain membrane integrity, buffer inflammatory responses, and support neuronal repair (7). Conversely, marked metabolic dysregulation—such as stress-induced hyperglycemia combined with low lipid levels—often reflects poor metabolic reserve and can amplify oxidative stress, compromise the blood–brain barrier, and exacerbate neuroinflammation, thereby increasing the likelihood of HT and unfavorable outcomes (8, 9). Given the tight interdependence between glucose and lipid pathways in acute ischemic stress responses, biomarkers that reflect their integrated metabolic status may provide superior insight into the body’s metabolic resilience and recovery potential compared with isolated parameters.

In this context, composite metabolic indicators have attracted increasing attention. Remnant cholesterol (RC), carried predominantly in triglyceride-rich lipoproteins such as intermediate-density lipoprotein (IDL) and very low-density lipoprotein (VLDL), has emerged as a promising surrogate marker and has been closely linked to atherosclerotic burden and cardiocerebrovascular events (10, 11). Additionally, the Cholesterol, high-density lipoprotein, and glucose (CHG) index, which incorporates total cholesterol (TC), HDL-C, and fasting blood glucose (FBG), provides a more integrated assessment of glucose–lipid metabolic balance (12). Prior research suggests that moderate CHG levels may reflect favorable metabolic reserve, anti-inflammatory capacity, and a more stable metabolic phenotype, and are associated with reduced cardiovascular risk (12, 13). Thus, CHG may not only indicate metabolic dysregulation but also capture potentially protective metabolic states during the acute phase of stroke.

Nevertheless, no study to date has investigated the roles of RC and CHG—or their potential synergistic effects—in predicting HT and functional outcomes after IV thrombolysis in AIS patients. Therefore, this dual-center cohort study aimed to systematically evaluate the independent and combined predictive value of RC and CHG for HT and 90-day outcomes following IV-rtPA, to provide novel metabolic biomarkers to improve early risk stratification and guide individualized management in hyperacute stroke care.

## 2 Methods

### 2.1 Study design

This multicenter, retrospective, observational study enrolled AIS patients who underwent intravenous rt-PA thrombolysis at the First Affiliated Hospital with Nanjing Medical University and the Affiliated Hospital of Xuzhou Medical University from January 2018 to October 2025. Inclusion criteria required meeting all of the following: (1) Age ≥18 years; (2) AIS confirmed by CT/MRI imaging; (3) Onset-to-treatment time window ≤4.5 hours; (4) Standard rt-PA intravenous thrombolysis protocol (total dose 0.9 mg/kg, maximum ≤90 mg: initial 10% bolus over 1 minute, remaining 90% infused over 60 minutes); Exclusion criteria included: (1) Patients receiving bridging therapy (intravenous thrombolysis combined with endovascular intervention or intra-arterial thrombolysis) (n= 53); (2) Severe cardiorenal dysfunction, coagulation disorders, history of substance abuse, or hemodynamic instability (n = 27); (3) Chronic liver/kidney failure or chronic heart failure (n = 14); (4) Systemic inflammatory/infectious diseases, hematological disorders, or immunosuppressive therapy within 2 weeks pre-admission (n = 78); (5) Comorbidities potentially affecting inflammatory biomarkers (malignancy, acute myocardial infarction, major trauma, recent surgery, or allergic diseases) (n = 45); (6) Incomplete hospitalization records or missing data impacting follow-up assessments (including missing components required to calculate RC/CHG, adjudicate HT, or determine 90-day mRS) (n = 129).

**Figure 1:**
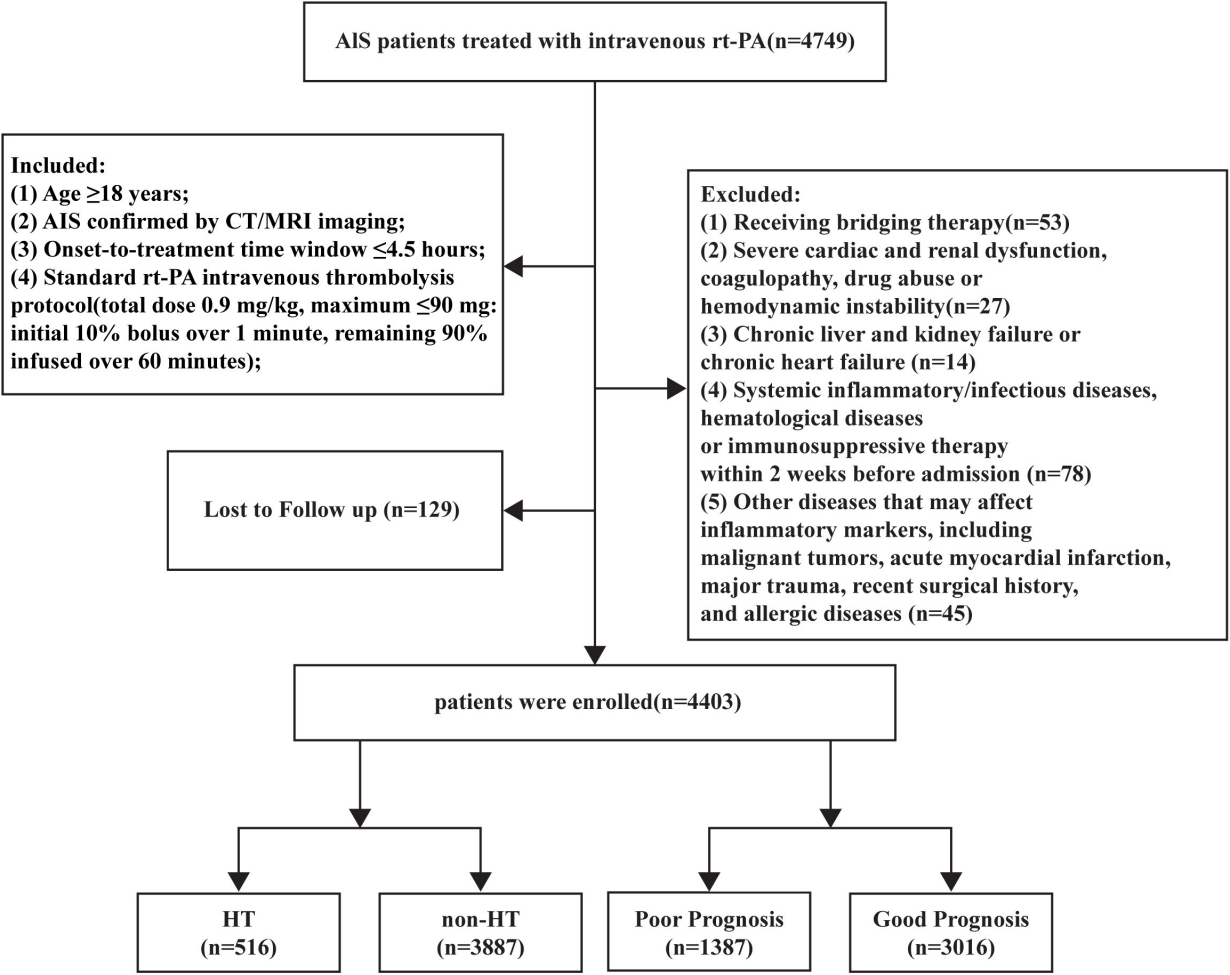
Flowchart of patient selection.

The study strictly adhered to the ethical principles of the Declaration of Helsinki and was approved by the Ethics Committees of the First Affiliated Hospital with Nanjing Medical University (Approval No.: 2025-SR-414) and the Affiliated Hospital of Xuzhou Medical University (Approval No.: XYFY2018-KL038-01). Informed consent was obtained from all participants or their legal representatives. The patient screening process is illustrated in **Figure 1**.

### 2.2 Data collection and definition

Comprehensive clinical information was obtained at admission, including demographic characteristics, conventional vascular risk profiles, medical comorbidities, infarct distribution (anterior or posterior circulation, or both), laboratory indicators, neuroimaging assessments, and in-hospital therapeutic interventions. Body mass index was derived from the ratio of body weight (kg) to height squared (m²) (14). The presence of hypertension, diabetes mellitus, atrial fibrillation, and coronary artery disease was determined according to diagnostic standards recommended by the World Health Organization. Etiologic subtypes of ischemic stroke were classified according to the Trial of Org 10172 in Acute Stroke Treatment (TOAST) criteria (15), including large-artery atherosclerosis (LAA), cardio-embolism (CE), small-artery occlusion (SAO), and others.

Peripheral venous blood was drawn within the first 24 hours after hospital arrival for hematologic testing, biochemical analysis, and coagulation profiling, in accordance with routine institutional protocols (16, 17). When several sets of laboratory values were available during this period, the earliest results were selected for analysis. RC was calculated indirectly from baseline lipid components using the equation: RC = TC − LDL-C − HDL-C (mmol/L). The CHG index was calculated using the formula: CHG index = ln [TC (mmol/L) × FBG (mmol/L)/2 × HDL-C (mmol/L)].

### 2.3 Outcome assessment

The primary outcome was HT, defined as any new intracranial hemorrhage confirmed by CT or MRI within 24 hours of intravenous thrombolysis, with no evidence of hemorrhage on pre-thrombolysis imaging (18, 19). The secondary outcome was the functional status at 3 months, evaluated using the modified Rankin Scale (mRS). Stroke-specialized neurologists performed follow-up assessments through standardized outpatient visits or structured telephone interviews. Functional outcomes were further categorized as good (mRS ≤ 2) or poor (mRS ≥ 3) (20).

### 2.4 Statistical analysis

The distribution of continuous variables was examined using the Shapiro–Wilk test. Normally distributed data were expressed as mean ± standard deviation and compared using independent-sample t-tests. In contrast, non-normally distributed variables were presented as median (interquartile range) and analyzed with the Mann–Whitney U test. Categorical data were summarized as counts and percentages and compared using Pearson’s χ² test or Fisher’s exact test as appropriate. RC and CHG were analyzed both as continuous variables and as quartile-based categories. Candidate variables associated with clinical outcomes in univariate analyses were further evaluated in multivariable logistic regression. Three models were constructed, guided by prior evidence and clinical relevance (21, 22): Model 1 (unadjusted), Model 2 (adjusted for age and sex), and Model 3 (additionally adjusted for smoking, drinking, hypertension, diabetes, atrial fibrillation, prior stroke/TIA, infarct distribution, baseline NIHSS score, systolic blood pressure, onset-to-needle time, antiplatelet and anticoagulant use, statin therapy, HbA1c and TOAST subtype).

To assess combined metabolic effects, a discordance analysis categorized patients into four groups according to the median RC and CHG values. Restricted cubic spline (RCS) models with four knots were employed to characterize potential nonlinear dose–response associations and identify threshold effects. Prespecified subgroup analyses were conducted across demographic and clinical categories to explore effect modification using stratified multivariable models. The predictive value of RC, CHG, and their combination for HT and 90-day outcomes was examined using receiver operating characteristic curves. Additionally, two other metrics, the net reclassification index (NRI) and integrated discrimination improvement (IDI), were calculated to assess the incremental predictive value of RC, CHG, and their combination. Mediation analyses based on the Baron–Kenny framework were used to determine whether the systemic immune-inflammation index (SII) mediated the associations of RC and CHG with study outcomes after full adjustment. All analyses were performed using SPSS 26.0, R 4.3.1, and MedCalc 20.1, with two-tailed *P* < 0.05 considered statistically significant.

## 3 Results

### 3.1 Baseline population characteristics

Of the 4403 included patients, 2850 (64.7%) were male and 1553 (35.3%) were female, with a median age of 68 years (IQR 59–76). The HT rate was 11.7% (516/4403). Additionally, 3016 (68.5%) had a good prognosis (three-month mRS ≤2), whereas 1387 (31.5%) had a poor prognosis (three-month mRS ≥3).

### 3.2 Baseline characteristics according to HT status

**Figure 2:**
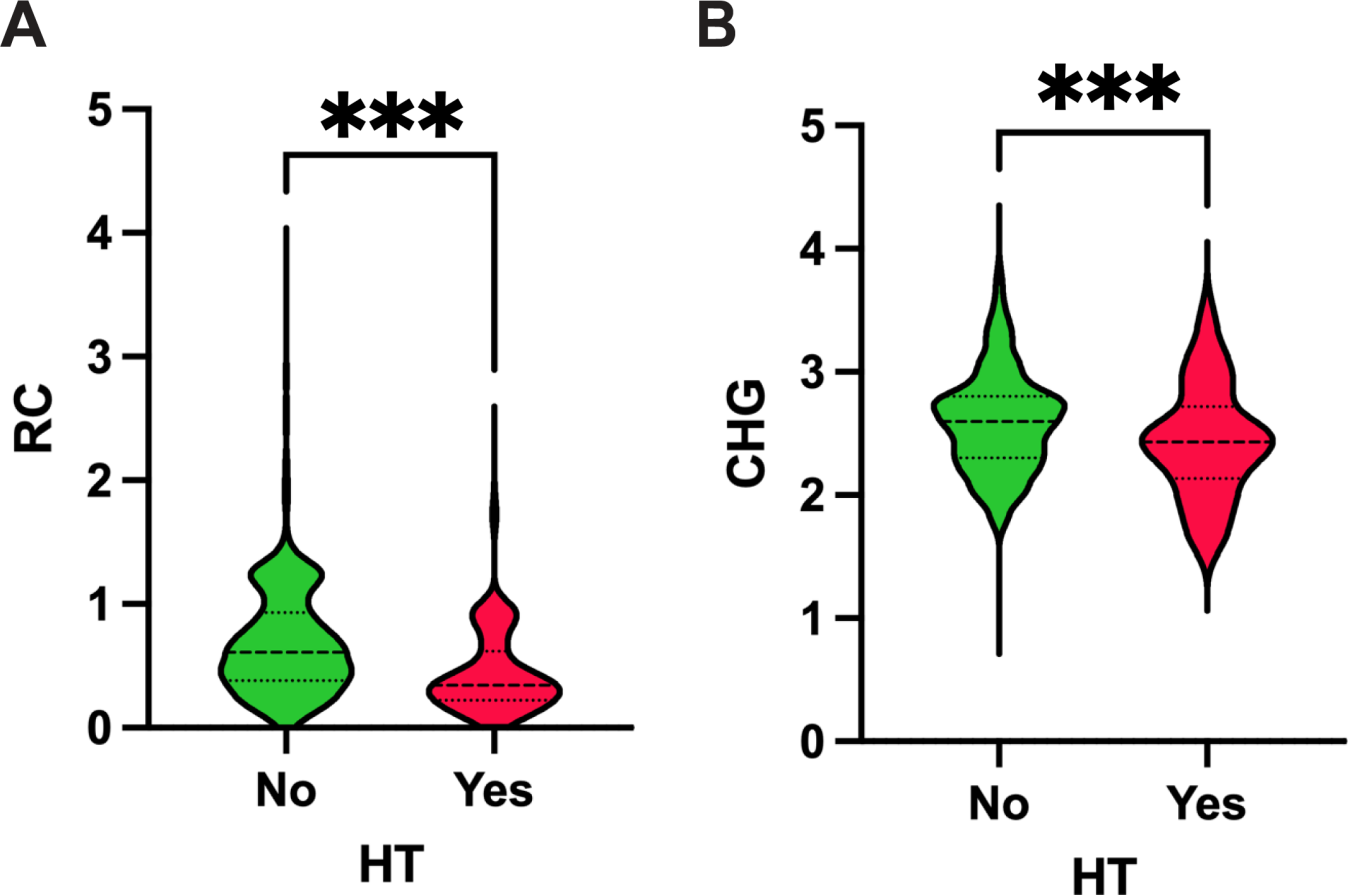
Violin plots showing the distribution of RC and CHG in patients with and without HT. ***P < 0.001.

Compared with the non-HT group, patients who developed HT were older and had higher baseline NIHSS scores, longer onset-to-needle times, and higher systolic and diastolic blood pressures (all *P* < 0.01). They also exhibited elevated homocysteine, HDL-C, HbA1c, and hs-CRP levels. HT patients were more likely to have atrial fibrillation, a history of smoking and drinking, and prior anticoagulant use, and they differed significantly in TOAST classification and infarct distribution. By contrast, the HT group had lower total cholesterol, triglycerides, LDL-C, RC, and CHG index values, as well as a lower prevalence of hypertension and prior use of antiplatelet agents, statins, or antihypertensives (all *P* < 0.05). (**Figure 2**; **Table 1**).

**Table 1:** Baseline characteristics between the HT and non-HT groups.

Variable	Overall (n=4403)	HT (n=516)	Non-HT (n=3887)	*P*
Demographic characteristics				
Male, n (%)	2850 (64.73)	337 (65.31)	2513 (64.65)	0.769
Age, years	68.00 (59.00, 76.00)	71.00 (62.00, 79.00)	68.00 (59.00, 76.00)	<0.001
BMI, kg/m^2^	24.90 (22.78, 27.18)	25.09 (22.44, 27.16)	24.83 (22.80, 27.18)	0.816
Risk factors, n (%)				
Smoking history	1078 (24.48)	146 (28.29)	932 (23.98)	0.032
Drinking history	1147 (26.05)	161 (31.20)	986 (25.37)	0.005
Hypertension	2957 (67.16)	289 (56.01)	2668 (68.64)	<0.001
Diabetes	1179 (26.78)	143 (27.71)	1036 (26.65)	0.609
Atrial fibrillation	804 (18.26)	214 (41.47)	590 (15.18)	<0.001
Previous stroke or TIA	1213 (27.55)	122 (23.64)	1091 (28.07)	0.035
CHD	722 (16.40)	94 (18.22)	628 (16.16)	0.235
Medical history, n (%)				
Prior antiplatelet	508 (11.54)	38 (7.36)	470 (12.09)	0.002
Prior anticoagulant	86 (1.95)	25 (4.84)	61 (1.57)	<0.001
Prior statin	401 (9.11)	26 (5.04)	375 (9.65)	0.001
Antihypertensive	1548 (35.16)	154 (29.84)	1394 (35.86)	0.007
Prior hypoglycemic	612 (13.90)	71 (13.76)	541 (13.92)	0.922
Clinical variables				
NIHSS, score	7.00 (4.00, 12.00)	15.00 (9.00, 21.00)	6.00 (4.00, 11.00)	<0.001
ONT, min	165.00 (111.00, 219.00)	185.00 (133.00, 240.00)	164.00 (111.00, 215.00)	<0.001
SBP, mmHg	152.00 (139.00, 168.00)	157.00 (143.00, 168.00)	151.00 (139.00, 168.00)	0.001
DBP, mmHg	86.00 (78.00, 96.00)	87.00 (79.00, 99.00)	86.00 (78.00, 95.00)	<0.001
Infarct distribution, n (%)				<0.001
Anterior circulation	2248 (51.06)	226 (43.80)	2022 (52.02)	
Posterior circulation	1160 (26.35)	59 (11.43)	1101 (28.33)	
Anterior and posterior circulation	995 (22.60)	231 (44.77)	764 (19.66)	
TOAST, n (%)				<0.001
LAA	1814 (41.20)	277 (53.68)	1537 (39.54)	
CE	555 (12.61)	152 (29.46)	403 (10.37)	
SAO	1663 (37.77)	47 (9.11)	1616 (41.57)	
Others	371 (8.43)	40 (7.75)	331 (8.52)	
Laboratory data				
FBG, mmol/L	6.34 (5.39, 7.11)	5.97 (5.26, 8.72)	6.39 (5.40, 7.06)	0.100
HbA1c, %	6.20 (5.60, 6.60)	6.57 (5.80, 7.10)	6.10 (5.60, 6.60)	<0.001
Homocysteine, umol/L	16.28 (12.40, 17.50)	17.04 (13.50, 17.04)	15.99 (12.32, 17.69)	<0.001
Total cholesterol (mmol/L)	4.41 (3.69, 5.03)	4.10 (3.30, 4.73)	4.46 (3.73, 5.05)	<0.001
Triglycerides (mmol/L)	1.34 (0.97, 1.71)	1.19 (0.82, 1.48)	1.37 (0.98, 1.75)	<0.001
LDL-C, mmol/L	2.66 (2.15, 3.12)	2.66 (1.85, 2.99)	2.66 (2.17, 3.16)	<0.001
HDL, mmol/L	1.09 (0.93, 1.22)	1.15 (1.00, 1.24)	1.09 (0.92, 1.22)	<0.001
hs-CRP, mg/L	2.20 (0.60, 7.10)	4.10 (0.90, 13.00)	2.00 (0.60, 6.10)	<0.001
RC	0.58 (0.34, 0.89)	0.35 (0.23, 0.62)	0.61 (0.38, 0.93)	<0.001
CHG	2.58 (2.29, 2.80)	2.43 (2.14, 2.71)	2.60 (2.30, 2.80)	<0.001

HT, hemorrhagic transformation; BMI, Body Mass Index; TIA, transient ischemic attack; CHD, Coronary heart disease; NIHSS, National Institutes of Health Stroke Scale; ONT, Onset-to-Needle Time; SBP, systolic blood pressure; DBP, diastolic blood pressure; TOAST, Trial of Org 10,172 in Acute Stroke Treatment; LAA, large-artery atherosclerosis; CE, cardio-embolism; SAO, small-artery occlusion; FBG, Fasting blood glucose; LDL, low-density lipoprotein; HDL, high-density lipoprotein; hs-CRP, hyper-sensitive C-reactive protein; RC, remnant cholesterol; CHG, Cholesterol-High-Density Lipoprotein Glucose index;

### 3.3 Baseline characteristics according to 3-month prognosis

**Figure 3:**
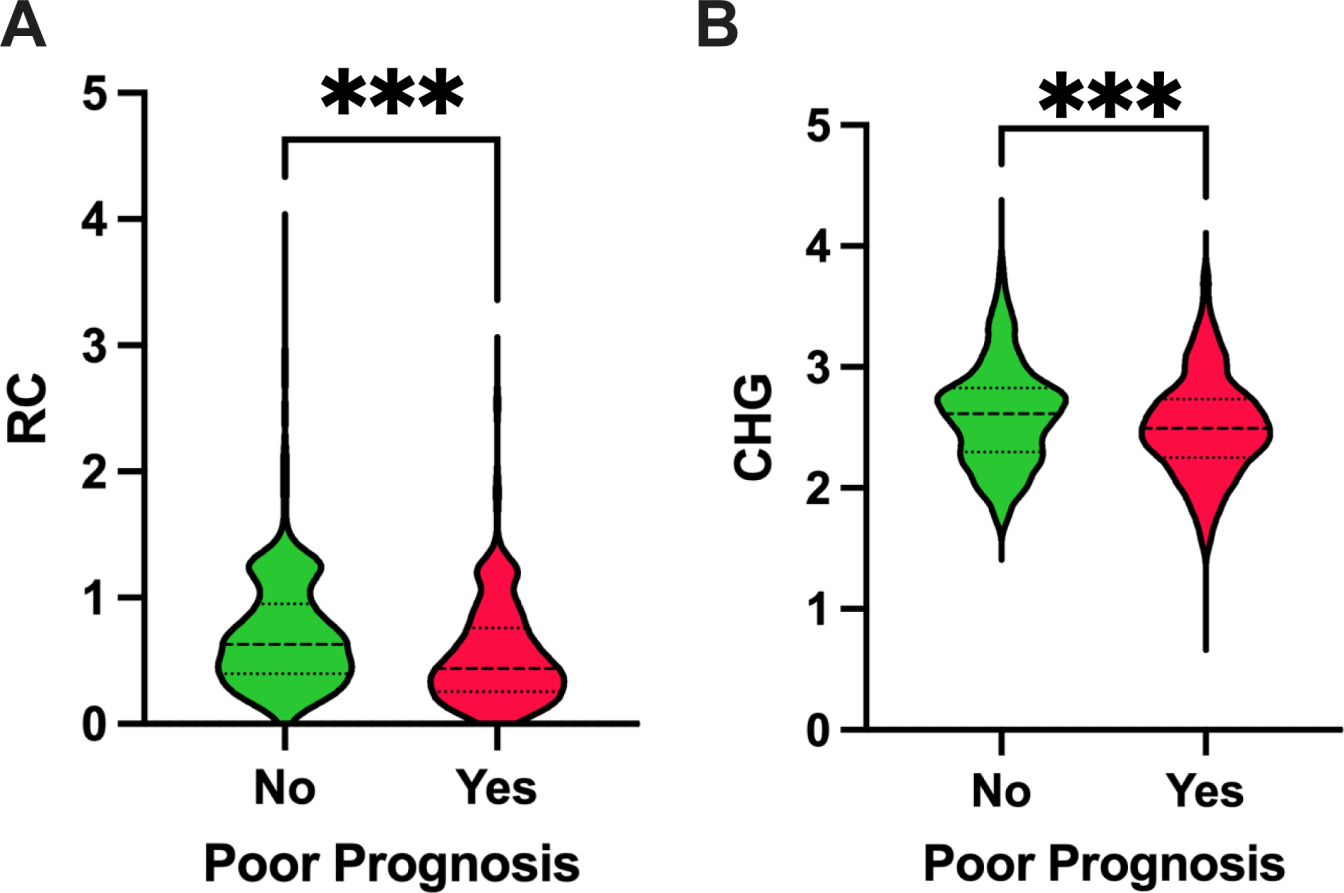
Violin plots demonstrating RC and CHG distribution in favorable and unfavorable prognosis groups. ***P < 0.001.

Compared with the good-prognosis group, the poor-prognosis group was older and had higher baseline NIHSS scores, systolic blood pressure, FBG, HbA1c, hs-CRP, and HDL-C levels, but lower BMI, TC, triglycerides, LDL-C, RC, and CHG index (all *P* < 0.05). They more frequently had hypertension, atrial fibrillation, a history of previous stroke or TIA, and prior anticoagulant or statin use, whereas smoking was less common. Significant differences were also observed in TOAST classification and infarct distribution between the two groups (all *P* < 0.05). (**Figure 3**; **Table 2**).

**Table 2:** Baseline characteristics between the poor and good prognosis groups.

Variable	Overall (n=4403)	Poor prognosis (n=1387)	Good prognosis (n=3016)	*P*
Demographic characteristics				
Male, n (%)	2850 (64.73)	869 (62.65)	1981 (65.68)	0.051
Age, years	68.00 (59.00, 76.00)	72.00 (63.00, 81.00)	67.00 (58.00, 74.00)	<0.001
BMI, kg/m^2^	24.90 (22.78, 27.18)	24.36 (21.78, 26.23)	25.18 (22.92, 27.44)	<0.001
Risk factors, n (%)				
Smoking history	1078 (24.48)	302 (21.77)	776 (25.73)	0.005
Drinking history	1147 (26.05)	338 (24.37)	809 (26.82)	0.085
Hypertension	2957 (67.16)	974 (70.22)	1983 (65.75)	0.003
Diabetes	1179 (26.78)	359 (25.88)	820 (27.19)	0.364
Atrial fibrillation	804 (18.26)	373 (26.89)	431 (14.29)	<0.001
Previous stroke or TIA	1213 (27.55)	504 (36.34)	709 (23.51)	<0.001
CHD	722 (16.40)	238 (17.16)	484 (16.05)	0.355
Medical history, n (%)				
Prior antiplatelet	508 (11.54)	171 (12.33)	337 (11.17)	0.265
Prior anticoagulant	86 (1.95)	48 (3.46)	38 (1.26)	<0.001
Prior statin	401 (9.11)	144 (10.38)	257 (8.52)	0.046
Antihypertensive	1548 (35.16)	474 (34.17)	1074 (35.61)	0.354
Prior hypoglycemic	612 (13.90)	205 (14.78)	407 (13.49)	0.252
Clinical variables				
NIHSS, score	7.00 (4.00, 12.00)	12.00 (8.00, 17.00)	6.00 (4.00, 9.00)	<0.001
ONT, min	165.00 (111.00, 219.00)	169.00 (115.00, 225.00)	164.00 (111.00, 215.00)	0.143
SBP, mmHg	152.00 (139.00, 168.00)	159.00 (143.00, 170.00)	150.00 (137.00, 165.00)	<0.001
DBP, mmHg	86.00 (78.00, 96.00)	86.00 (79.00, 95.00)	85.00 (78.00, 96.00)	0.137
Infarct distribution, n (%)				<0.001
Anterior circulation	2248 (51.06)	644 (46.43)	1604 (53.18)	
Posterior circulation	1160 (26.35)	355 (25.59)	805 (26.69)	
Anterior and posterior circulation	995 (22.60)	388 (27.97)	607 (20.13)	
TOAST, n (%)				<0.001
LAA	1814 (41.20)	677 (48.81)	1137 (37.70)	
CE	555 (12.61)	265 (19.11)	290 (9.62)	
SAO	1663 (37.77)	352 (25.38)	1311 (43.47)	
Others	371 (8.43)	93 (6.71)	278 (9.22)	
Laboratory data				
FBG, mmol/L	6.34 (5.39, 7.11)	6.35 (5.46, 7.68)	6.33 (5.36, 7.03)	0.005
HbA1c, %	6.20 (5.60, 6.60)	6.30 (5.70, 6.60)	6.10 (5.60, 6.68)	0.005
Homocysteine, umol/L	16.28 (12.40, 17.50)	16.85 (12.66, 17.12)	16.10 (12.30, 17.68)	0.150
Total cholesterol (mmol/L)	4.41 (3.69, 5.03)	4.28 (3.58, 4.91)	4.47 (3.74, 5.07)	<0.001
Triglycerides (mmol/L)	1.34 (0.97, 1.71)	1.19 (0.88, 1.49)	1.40 (1.01, 1.85)	<0.001
LDL-C, mmol/L	2.66 (2.15, 3.12)	2.66 (2.03, 2.99)	2.66 (2.18, 3.21)	<0.001
HDL, mmol/L	1.09 (0.93, 1.22)	1.15 (1.00, 1.28)	1.07 (0.90, 1.19)	<0.001
hs-CRP, mg/L	2.20 (0.60, 7.10)	3.20 (0.60, 8.80)	2.00 (0.55, 5.30)	<0.001
RC	0.58 (0.34, 0.89)	0.44 (0.26, 0.76)	0.63 (0.40, 0.95)	<0.001
CHG	2.58 (2.29, 2.80)	2.49 (2.25, 2.74)	2.61 (2.30, 2.83)	<0.001

HT, hemorrhagic transformation; BMI, Body Mass Index; TIA, transient ischemic attack; CHD, Coronary heart disease; NIHSS, National Institutes of Health Stroke Scale; ONT, Onset-to-Needle Time; SBP, systolic blood pressure; DBP, diastolic blood pressure; TOAST, Trial of Org 10,172 in Acute Stroke Treatment; LAA, large-artery atherosclerosis; CE, cardio-embolism; SAO, small-artery occlusion; FBG, Fasting blood glucose; LDL, low-density lipoprotein; HDL, high-density lipoprotein; hs-CRP, hyper-sensitive C-reactive protein; RC, remnant cholesterol; CHG, Cholesterol-High-Density Lipoprotein Glucose index.

### 3.4 Associations of RC and CHG with HT and poor prognosis

In multivariable logistic regression analyses, both RC and the CHG index showed a significant negative association with HT and poor prognosis, whether evaluated as continuous measures or categorical quartiles. In the fully adjusted model (Model 3), higher RC and CHG levels were significantly associated with decreased HT risk (RC: OR = 0.27, 95% CI: 0.19–0.37; CHG: OR = 0.34, 95% CI: 0.24–0.47; both *P* < 0.001). Quartile analyses demonstrated consistent patterns, showing substantially lower HT likelihood in the highest quartiles of RC (adjusted OR = 0.22, 95% CI: 0.16–0.29) and CHG (adjusted OR = 0.58, 95% CI: 0.45–0.74), with significant linear trends. Similar associations were observed for functional outcomes: each unit increase in RC and CHG was associated with a reduced risk of poor prognosis (RC: OR = 0.39, 95% CI: 0.31–0.48; CHG: OR = 0.46, 95% CI: 0.36–0.58), and quartile analyses confirmed markedly lower risk in their highest categories (RC: adjusted OR = 0.37, CHG: adjusted OR = 0.65; both *P* for trend < 0.001). (**Table 3**).

**Table 3:** Associations of RC and CHG with HT and poor prognosis.

Variables		Model 1		Model 2		Model 3	
		OR (95%CI)	*P*	OR (95%CI)	*P*	OR (95%CI)	*P*
HT							
RC	Continuous	0.16 (0.12-0.22)	<0.001	0.17 (0.12-0.23)	<0.001	0.27 (0.19-0.37)	<0.001
	Q1	*Ref* (1.00)		*Ref* (1.00)		*Ref* (1.00)	
	Q2	0.46 (0.36-0.57)	<0.001	0.47 (0.37-0.59)	<0.001	0.45 (0.36-0.57)	<0.001
	Q3	0.22 (0.16-0.29)	<0.001	0.23 (0.17-0.30)	<0.001	0.23 (0.17-0.30)	<0.001
	Q4	0.22 (0.16-0.29)	<0.001	0.23 (0.17-0.31)	<0.001	0.22 (0.16-0.29)	<0.001
	*P* for trend	—	<0.001	—	<0.001	—	<0.001
CHG	Continuous	0.39 (0.31-0.49)	<0.001	0.42 (0.33-0.53)	<0.001	0.34 (0.24-0.47)	<0.001
	Q1	*Ref* (1.00)		*Ref* (1.00)		*Ref* (1.00)	
	Q2	0.91 (0.73-1.14)	0.420	0.95 (0.75-1.19)	0.623	0.90 (0.72-1.13)	0.370
	Q3	0.18 (0.12-0.25)	<0.001	0.18 (0.12-0.26)	<0.001	0.16 (0.11-0.23)	<0.001
	Q4	0.59 (0.46-0.75)	<0.001	0.65 (0.50-0.83)	0.001	0.58 (0.45-0.74)	<0.001
	*P* for trend	—	<0.001	—	<0.001	—	<0.001
Poor prognosis							
RC	Continuous	0.36 (0.31-0.43)	<0.001	0.41 (0.34-0.49)	<0.001	0.39 (0.31-0.48)	<0.001
	Q1	*Ref* (1.00)		*Ref* (1.00)		*Ref* (1.00)	
	Q2	0.62 (0.53-0.74)	<0.001	0.68 (0.57-0.81)	<0.001	0.63 (0.53-0.74)	<0.001
	Q3	0.41 (0.34-0.49)	<0.001	0.46 (0.38-0.55)	<0.001	0.41 (0.34-0.49)	<0.001
	Q4	0.37 (0.31-0.45)	<0.001	0.43 (0.36-0.52)	<0.001	0.37 (0.31-0.44)	<0.001
	*P* for trend	—	<0.001	—	<0.001	—	<0.001
CHG	Continuous	0.55 (0.47-0.64)	<0.001	0.65 (0.55-0.76)	<0.001	0.46 (0.36-0.58)	<0.001
	Q1	*Ref* (1.00)		*Ref* (1.00)		*Ref* (1.00)	
	Q2	1.22 (1.03-1.45)	0.025	1.33 (1.11-1.59)	0.002	1.23 (1.03-1.46)	0.021
	Q3	0.65 (0.54-0.78)	<0.001	0.70 (0.58-0.84)	<0.001	0.65 (0.54-0.78)	<0.001
	Q4	0.65 (0.54-0.78)	<0.001	0.80 (0.66-0.96)	0.018	0.65 (0.54-0.78)	<0.001
	*P* for trend	—	<0.001	—	<0.001	—	<0.001

Model 1, unadjusted; Model 2, adjusted for age and sex; and Model 3, further adjusted for smoking, drinking, hypertension, diabetes, atrial fibrillation, prior stroke or TIA, infarct distribution, baseline NIHSS score, SBP, ONT, antiplatelet therapy, anticoagulant therapy, statin use, HbA1c, and TOAST classification.

**Figure 4:**
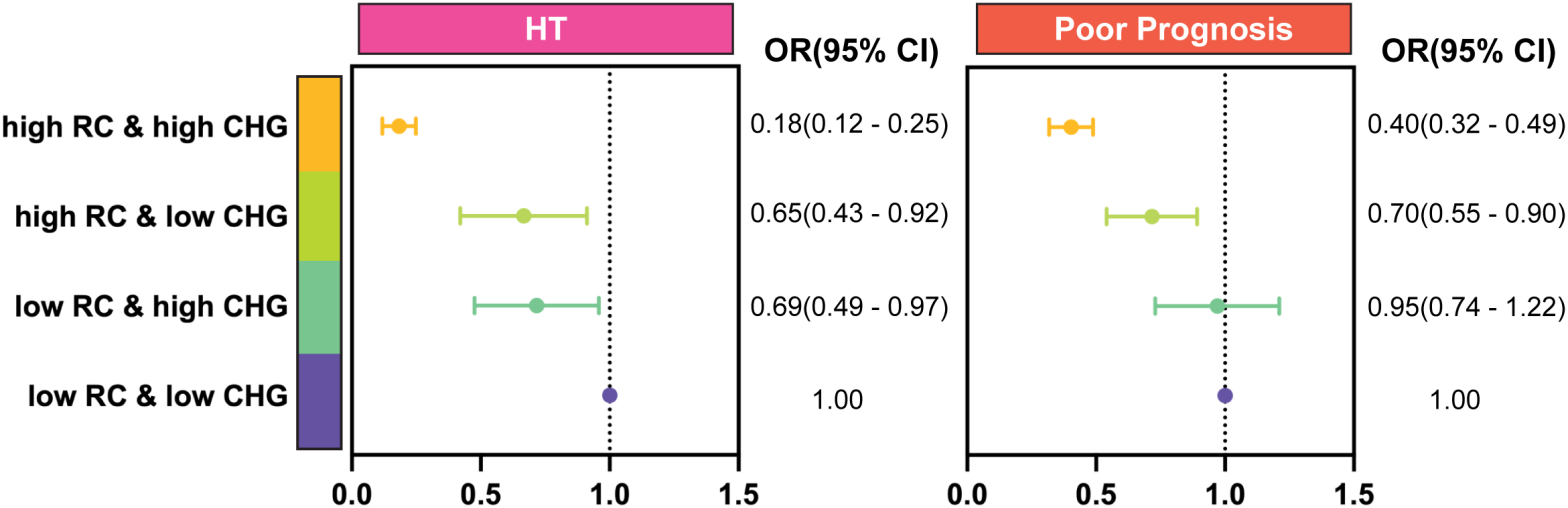
Discordance analyses of RC and CHG levels. The four groups (from bottom to top) were: Low RC and Low CHG group (serving as the baseline for comparison); Low RC and High CHG group (focusing on the effect of elevated CHG in the context of low RC levels); High RC and Low CHG group (focusing on the effect of elevated RC in the context of low CHG levels); High RC and High CHG group (focusing on the combined effect of both elevated RC and CHG levels on HT and poor prognosis. Odds ratios were calculated after adjustment for Model 3 covariates.

The discordance analysis further highlighted the joint protective effects of RC and CHG. Using median cut-offs (RC: 0.58 mmol/L; CHG: 2.58 mmol/L), patients with simultaneously elevated RC and CHG had the most significant reduction in HT risk (OR = 0.18, 95% CI: 0.12–0.25) compared with the low-RC/low-CHG reference group. Protective associations were also observed in the low-RC/high-CHG (OR = 0.69, 95% CI: 0.49–0.97) and high-RC/low-CHG (OR = 0.65, 95% CI: 0.43–0.92) groups. A similar pattern emerged for functional outcomes, in which the high-RC/low-CHG (OR = 0.70, 95% CI: 0.55–0.90) and high-RC/high-CHG (OR = 0.40, 95% CI: 0.32–0.49) groups exhibited significantly reduced odds of poor prognosis, whereas the low-RC/high-CHG group did not show a significant association. (**Figure 4**).

### 3.5 RCS analyses

**Figure 5:**
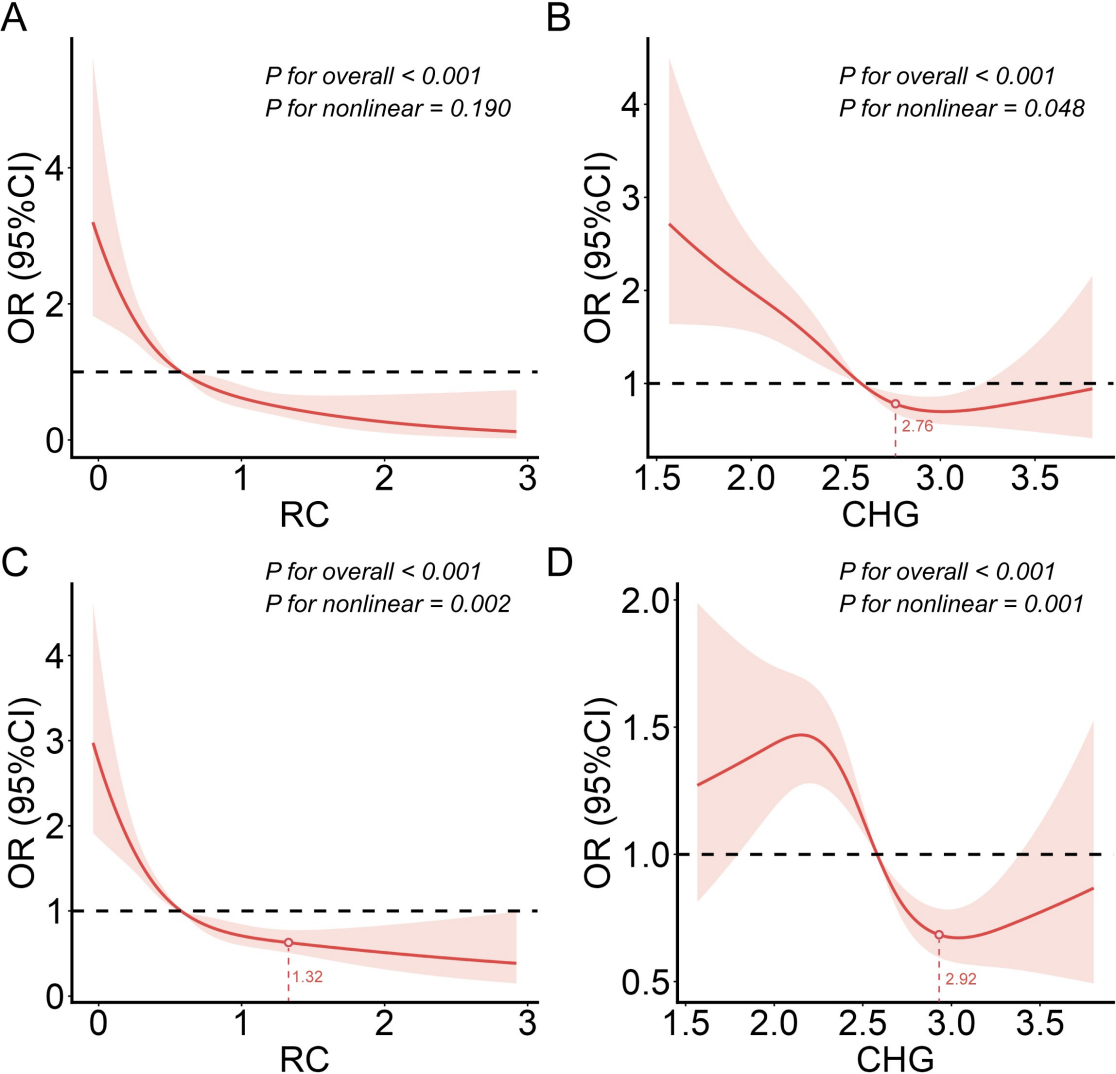
Restricted cubic spline (RCS) curves demonstrating the dose–response relationships between **(A)** RC and the risk of HT, **(B)** CHG and the risk of HT, **(C)** RC and the risk of poor prognosis, and **(D)** CHG and the risk of poor prognosis, after adjustment for Model 3 covariates.

Restricted cubic spline (RCS) analyses based on the fully adjusted Model 3 showed different dose–response associations for RC and CHG with HT and poor prognosis (**Figure 5**). RC demonstrated a significant overall association with HT following a linear inverse pattern (*P* for overall < 0.001; *P* for nonlinearity = 0.190), indicating a steady decline in HT risk with increasing RC and no evidence of threshold effects. By contrast, CHG exhibited a nonlinear association with HT, with a significant inflection point at CHG = 2.76. Below this threshold, higher CHG levels were associated with a substantially lower risk of HT (OR = 0.30, 95% CI: 0.19–0.45), whereas CHG values above 2.76 were linked to increased HT risk (OR = 1.16, 95% CI: 1.53–9.98; both *P* values < 0.01).

Nonlinear associations were similarly observed for both RC and CHG with poor prognosis (both *P* for overall < 0.001; *P* for nonlinearity < 0.01). For RC, the risk of poor outcome decreased sharply with rising concentrations up to an inflection point at 1.32; below this value, RC was strongly protective (OR = 0.29, 95% CI: 0.23–0.37), whereas RC levels above 1.32 were associated with increased risk (OR = 1.98, 95% CI: 3.23–13.49). CHG also showed a nonlinear pattern, with an inflection point at 2.92. When CHG < 2.92, higher levels predicted lower risk (OR = 0.45, 95% CI: 0.33–0.60), while CHG > 2.92 reversed the association (OR = 1.17, 95% CI: 1.16–5.79).

### 3.6 Subgroup analysis

**Figure 6:**
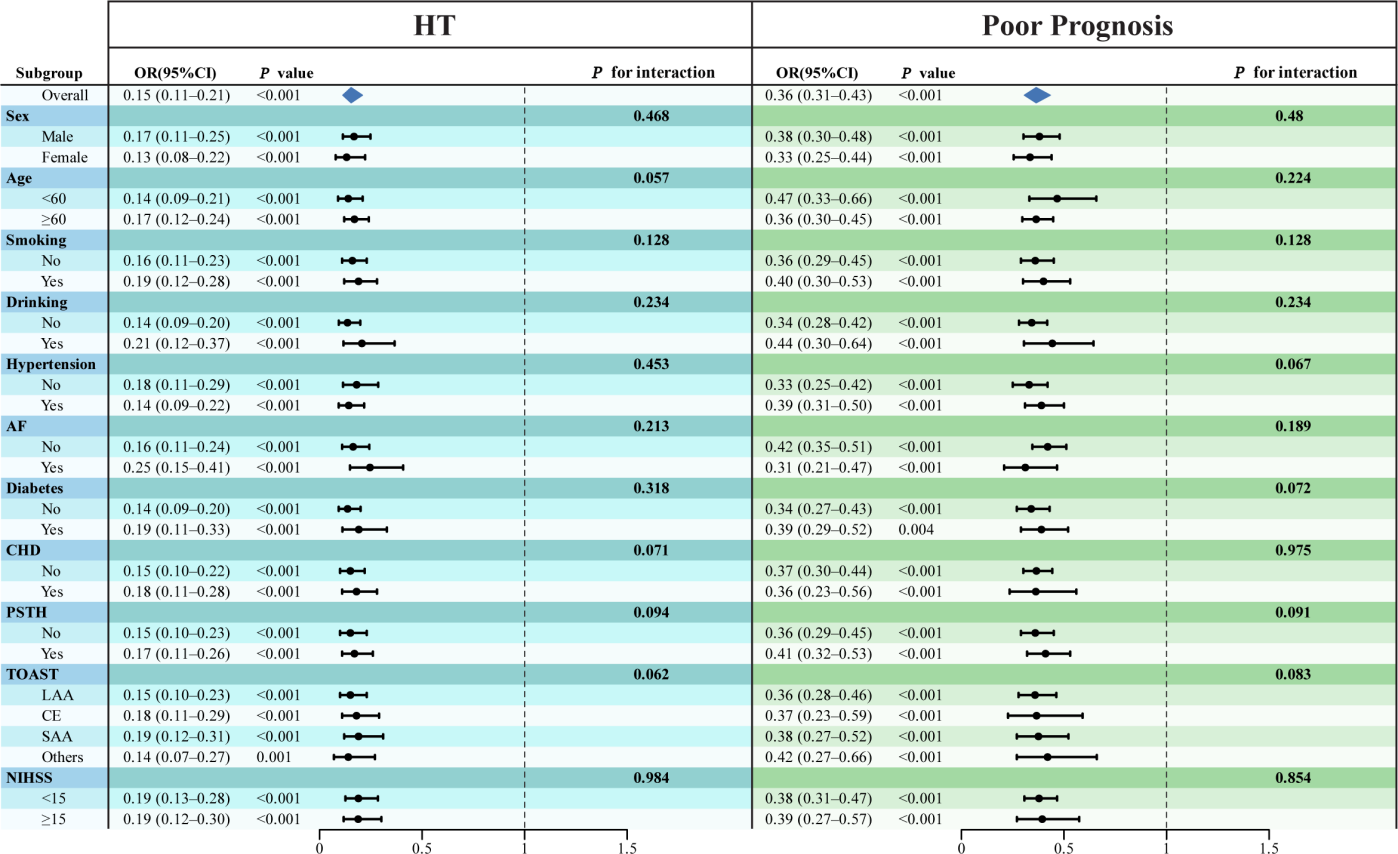
Subgroup analyses examining the relationship between RC and HT, poor prognosis in AIS, adjusted for Model 3 covariates.

Stratified multivariate regression analyses were conducted to further explore the associations of RC and CHG with the risks of HT and poor prognosis across strata of sex, age, smoking, drinking, hypertension, atrial fibrillation (AF), diabetes mellitus, coronary heart disease (CHD), history of prior stroke or TIA (PSTH), baseline NIHSS score, and TOAST classification (**Figure 6**; **Supplementary Figure S1**). As shown in **Figure 6**, the negative associations between RC and both HT and poor prognosis remained generally consistent across most subgroups after adjustment for Model 3 covariates (all *P* for interaction > 0.05). Similarly, as shown in **Supplementary Figure 1**, the associations between CHG and both HT and poor prognosis were generally consistent across most subgroups, with no significant effect modification observed (*P* for interaction > 0.05 for most comparisons). However, interactions were found for sex (*P* < 0.05) and hypertension (*P* < 0.001).

The joint associations of RC and CHG with HT and poor prognosis are summarized in **Supplementary Tables S3**, **S4**. Patients were classified into four groups based on the median RC (0.58 mmol/L) and CHG (2.58 mmol/L) levels: group 1 (low RC & low CHG, reference), group 2 (low RC & high CHG), group 3 (high RC & low CHG), and group 4 (high RC & high CHG). Compared with the reference group, patients in group 4 (high RC & high CHG) consistently had the lowest risk of HT and the most favorable prognosis across almost all subgroups (both *P* for trend < 0.001).

### 3.7 Prognostic value of RC, CHG, and their combination

**Figure 7:**
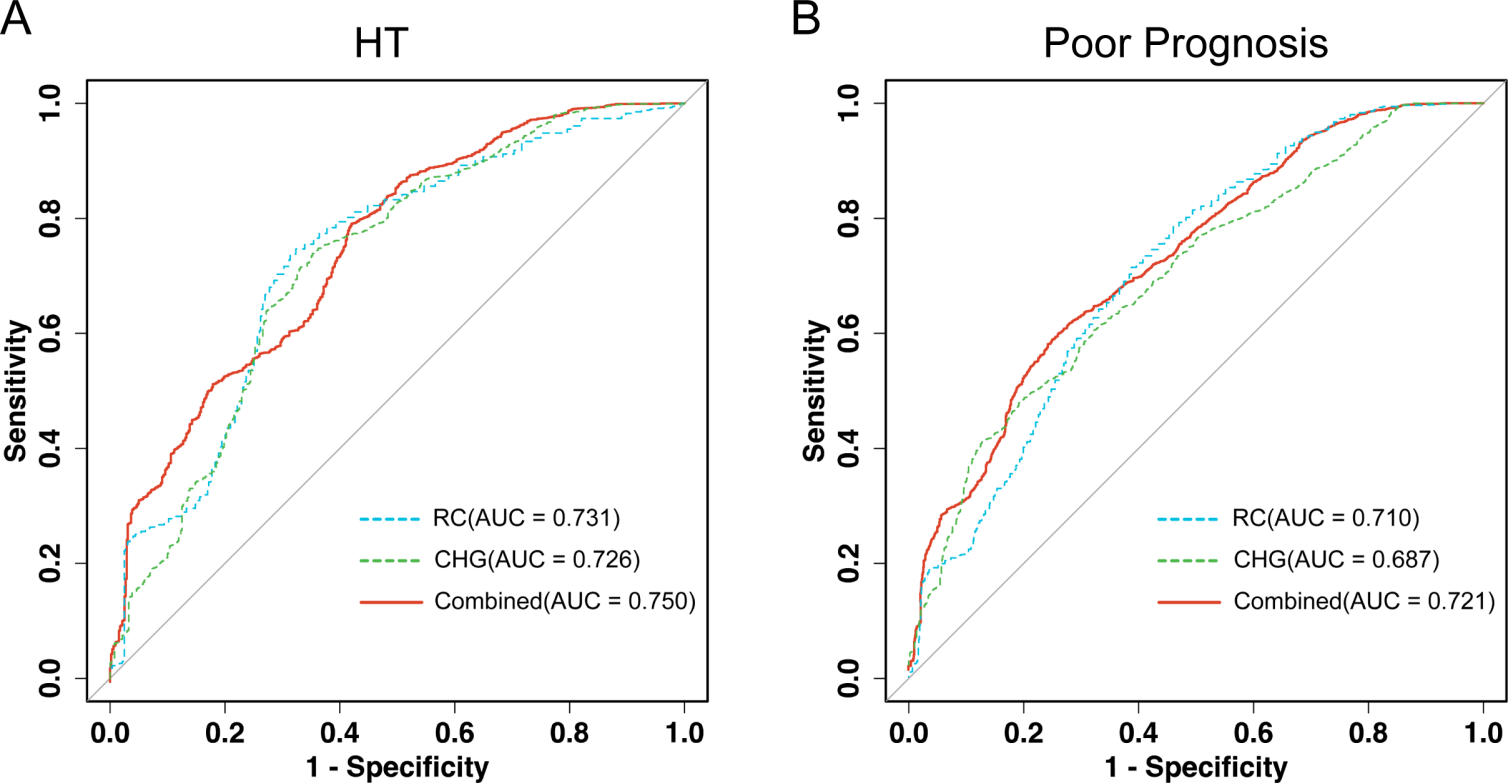
ROC curve analysis of RC, CHG, and their combination for predicting HT **(A)** and poor prognosis **(B)** after intravenous thrombolysis.

The ROC curves for RC, CHG, and their combination for HT and poor-prognosis prediction are shown in **Figure 7**. As illustrated in **Figure 7A**, the combination of them had a better predictive efficacy for HT than RC (AUC: 0.750 vs. 0.731, *P* = 0.039) or CHG (AUC: 0.750 vs. 0.726, *P* < 0.001) alone. Based on the maximum Youden index, the optimal cutoff values were 0.42 for RC, 2.39 for CHG, and 0.82 for the combined model, with corresponding sensitivity and specificity summarized in **Supplementary Table 5**. Meanwhile, to predict poor prognosis (**Figure 7B**), the AUC of the combined model was superior to RC (0.721 vs. 0.710, *P* = 0.028) and CHG (0.721 vs. 0.687, *P* < 0.001). The optimal cutoff values were 0.49 for RC, 2.76 for CHG, and 0.60 for the combined model, with detailed diagnostic performance indicators (AUC, sensitivity, and specificity) provided in **Supplementary Table 6**.

Furthermore, the incremental predictive value of RC, CHG, and their combination beyond the basic model was evaluated using the net reclassification improvement (NRI) and integrated discrimination improvement (IDI). As shown in **Supplementary Table 7**, adding RC or CHG to the basic model significantly improved risk reclassification and discrimination for HT. Notably, the combined model provided the greatest improvement (NRI: 0.610, 95% CI: 0.532–0.690; IDI: 0.097, 95% CI: 0.082–0.113; both *P* < 0.001).

Similarly, for predicting poor prognosis, the inclusion of RC, CHG, or their combination significantly enhanced the predictive performance of the basic model. The combined model achieved the largest improvement (NRI: 0.330, 95% CI: 0.270–0.390; IDI: 0.042, 95% CI: 0.035–0.048; both *P* < 0.001), suggesting that the joint assessment of RC and CHG provides additional prognostic value beyond conventional clinical risk factors.

### 3.8 Mediation analysis of SII in the associations of RC and CHG with HT and poor prognosis

**Figure 8:**
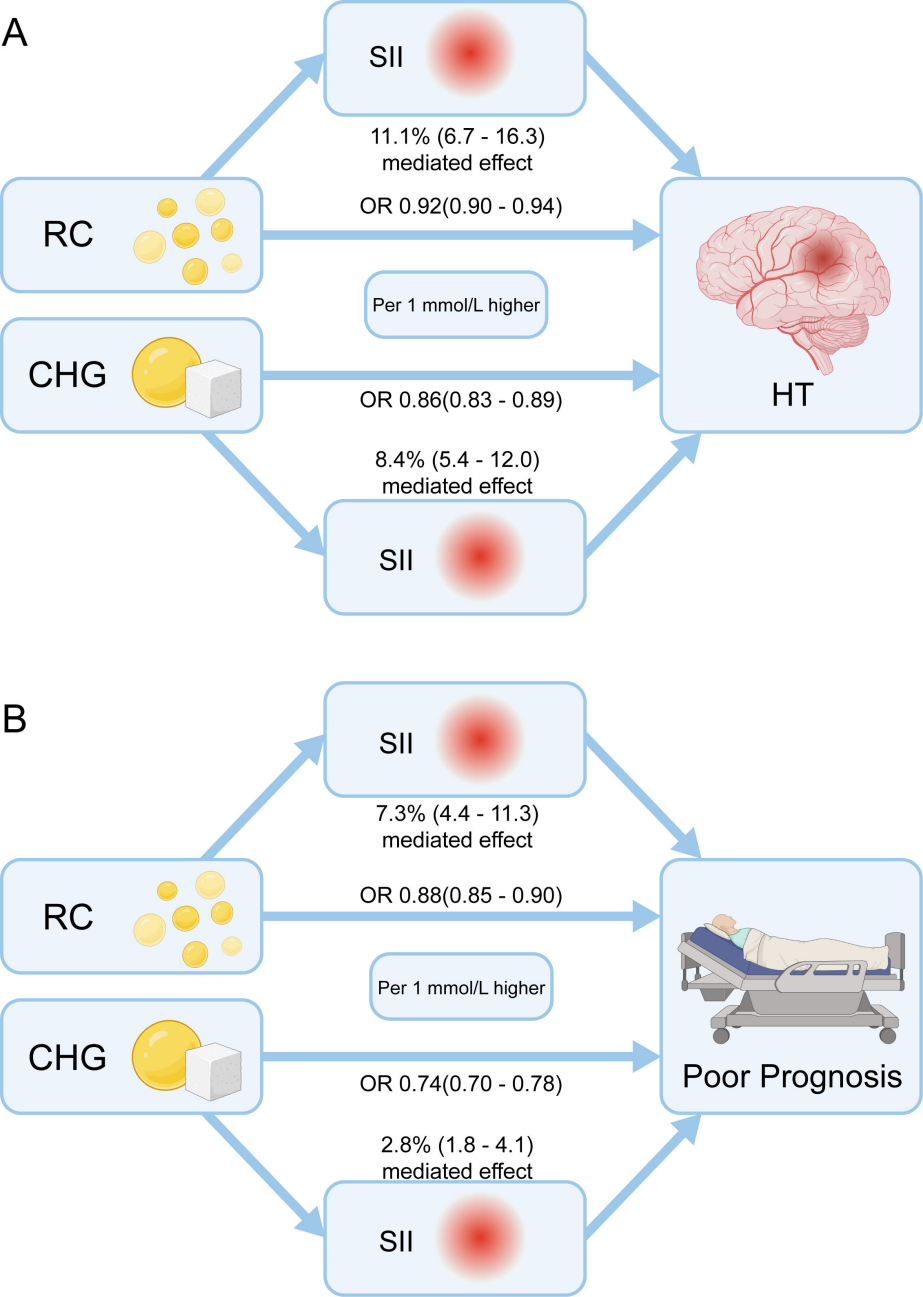
Mediation analysis of the role of SII in the associations between RC, CHG, and the risks of HT and poor prognosis. Mediation models showing the proportion of the effect of RC and CHG on HT and poor prognosis that is mediated by SII. All models were adjusted for Model 3 covariates.

Mediation analysis revealed that SII partially mediated the relationships between RC, CHG, and both HT and poor prognosis (**Figure 8**). Specifically, SII accounted for 11.1% (95% CI: 6.7–16.3%, *P* < 0.001) of the association between RC and HT. After adjusting for Model 3 covariates, each 1 mmol/L increase in RC was associated with a lower risk of HT (OR = 0.92, 95% CI: 0.90–0.94, *P* < 0.001). For CHG, the direct effect on HT yielded an OR of 0.86 (95% CI: 0.83–0.89, *P* < 0.001), with SII mediating 8.4% (95% CI: 5.4–12.0%, *P* < 0.001) of this relationship.

Regarding poor prognosis, higher RC levels were negatively associated with risk (OR = 0.88, 95% CI: 0.85–0.90, *P* < 0.001), and 7.3% (95% CI: 4.4–11.3%, *P* < 0.001) of this effect was mediated by SII. Similarly, each 1 mmol/L increase in CHG was associated with an OR of 0.74 (95% CI: 0.70–0.78, *P* < 0.001) for poor prognosis, with SII mediating 2.8% (95% CI: 1.8–4.1%, *P* < 0.001) of the effect.

Collectively, these findings indicate that systemic inflammation, as reflected by elevated SII, serves as a partial biological pathway linking lipid–glucose metabolic markers (RC and CHG) to both HT and poor prognosis following intravenous thrombolysis in AIS.

## Discussion

In this two-center retrospective cohort study, we demonstrated that elevated levels of RC and CHG—either individually or in combination—were independently associated with a reduced risk of HT and poor prognosis following intravenous thrombolysis in patients with AIS. Moreover, their combined use substantially improved predictive accuracy, underscoring their potential as easily accessible and multidimensional biomarkers for individualized risk stratification in clinical practice.

Previous studies have consistently reported that HT is one of the most severe complications following thrombolysis and is strongly associated with poor functional outcomes and increased mortality (23). However, its reported incidence varies markedly across cohorts, ranging from 10% to 48% (24). In our large sample (n = 4403), the incidence of HT was 11.7%, which is consistent with rates observed in randomized controlled trials, thereby supporting the robustness of our findings. Notably, our detailed analyses identified key nonlinear associations and revealed important mediating pathways involving SII.

RC is a major triglyceride-rich lipoprotein component originating primarily from IDL and VLDL. These particles can more easily penetrate the arterial wall and become trapped, directly contributing to atherosclerosis (5). Previous studies have shown that elevated RC levels are associated with a higher risk of myocardial infarction and ischemic stroke. Although the long-standing clinical principle that “lower cholesterol is better” remains foundational in lipid-lowering therapy for ischemic stroke (25), accumulating evidence suggests a lipid paradox—where higher cholesterol levels are associated with more favorable outcomes (5, 26). Earlier studies reported that higher cholesterol levels at stroke onset predicted better short-term functional recovery (27) and even improved 10-year survival (28).

Additionally, studies in acute coronary syndrome have shown that lower admission LDL-C levels predict worse long-term outcomes, independent of statin therapy at discharge (12). In line with this paradox, our study demonstrated that higher RC levels were associated with a reduced incidence of HT and better functional outcomes in patients with AIS. These findings support the notion that, in acute critical illness, lipid levels may reflect physiological reserves rather than solely long-term cardiovascular and cerebrovascular burden. Although the mechanisms underlying the lipid paradox remain uncertain, several plausible biological explanations exist. First, higher lipid levels may reflect greater nutritional or metabolic reserve, providing greater capacity to buffer physiological stress and support tissue repair during acute injury (26). Second, lipoproteins—particularly LDL-C and VLDL—can bind circulating endotoxins and inflammatory cytokines, thereby modulating innate immune responses (29). Third, cholesterol constitutes an essential structural component of neuronal membranes, and excessively low cholesterol levels may compromise cellular resilience under ischemic conditions. Cholesterol also serves as a precursor for stress-related hormones, such as cortisol, which participate in adaptive responses to acute injury (30). Finally, higher serum cholesterol levels have been associated with anti-inflammatory properties, including buffering against free radicals generated after ischemic attacks, which may enhance tolerance to hypoxia (31). Collectively, these mechanisms may explain why lipid profiles in acute settings often reflect overall physiological robustness rather than cardiovascular risk alone.

Our findings also revealed a distinct U-shaped, nonlinear association between CHG and the risk of HT and a poor prognosis, reflecting the dual metabolic implications of CHG across varying physiological states. This observation aligns with the results of Wei et al., who reported a similar U-shaped association between CHG and metabolic syndrome. As an index incorporating TC, FBG, and HDL-C, CHG captures integrated information on glucose and lipid metabolism. Cholesterol, together with glucose, plays a fundamental role in energy supply (12). Low CHG levels may reflect metabolic stress, malnutrition, chronic comorbidities, or infection, indicating impaired physiological reserve rather than health. Moderate levels of cholesterol and glucose, by contrast, support metabolic homeostasis and may confer metabolic protection in AIS (32). When CHG levels exceed the observed thresholds (2.76 or 2.92), such elevations often reflect pathological processes, including insulin resistance, chronic inflammation, and lipotoxicity (33, 34).

The additive effect observed between RC and CHG suggests a synergistic interaction between glucose and lipid metabolism. From a biological perspective, the combined assessment of RC and CHG may reflect the integrated regulation of lipid metabolism, glucose homeostasis, and systemic inflammation in AIS (35, 36). RC represents triglyceride-rich lipoproteins, whereas CHG captures broader metabolic disturbances related to insulin resistance and dyslipidemia. These metabolic alterations may contribute to endothelial dysfunction, oxidative stress, and blood–brain barrier instability, which are key processes involved in hemorrhagic transformation after thrombolysis (37, 38). In addition, lipoproteins may exert immunomodulatory effects by binding circulating endotoxins and inflammatory mediators, thereby attenuating excessive inflammatory responses (39). At the cellular level, disturbances in glucose and lipid metabolism may influence signaling pathways associated with ischemic injury, including oxidative stress responses, endothelial nitric oxide signaling, and inflammatory cascades such as NF-κB activation, ultimately affecting vascular integrity and reperfusion injury (40, 41). The consistent negative correlation between RC and HT, and the poor prognosis across all subgroups, further confirm the lipid paradox. Notably, a significant interaction was observed between CHG and both sex and hypertension. Compared with the non-hypertensive group, the negative correlation between CHG and HT and poor prognosis was weaker in the hypertensive group, consistent with previous studies (42). Furthermore, we found that the negative correlation of CHG was weaker in women than in men, possibly due to lower circulating levels of sex hormones in women at the onset of menopause, making them more susceptible to impaired insulin sensitivity and lipid dysregulation, which increases the risk of cardiovascular disease (43). Gong et al. found that the correlation between various IR indicators and metabolic diseases was more pronounced in women (44).

Our ROC analyses further supported the clinical relevance of RC and CHG as prognostic biomarkers and highlighted the advantage of their combined application. Although RC and CHG alone both demonstrated acceptable discriminative ability for predicting HT and poor prognosis (AUCs ranging from 0.687 to 0.731), the combined model consistently yielded higher AUCs for both endpoints, indicating superior predictive performance. Notably, the improvement in AUC, although modest in absolute magnitude, was statistically significant, suggesting that RC and CHG capture complementary metabolic information that cannot be fully reflected by either marker alone. This finding aligns with the concept that glucose and lipid metabolism are tightly interconnected in the acute ischemic state, and that their joint assessment provides a more comprehensive representation of metabolic resilience and vulnerability.

Moreover, our mediation analysis demonstrated that SII exerted an adverse mediating effect on the associations among RC, CHG, and stroke risk, further supporting the lipid paradox. Previous studies have shown that only about 5% of the excess risk of major adverse cardiovascular events (MACE) associated with RC is mediated through hsCRP, indicating that the pro-inflammatory effect of RC, as quantified by hsCRP, is unlikely to be the principal driver of increased cardiovascular risk (45). Although several large-scale observational and genetic studies have reported that both measured and genetically determined plasma RC levels are positively associated with higher hsCRP concentrations, reflecting an overall increase in systemic inflammatory status, our findings extend this evidence by showing a negative mediation between RC and the commonly used systemic composite inflammatory index SII (46, 47). This suggests that the relationship between RC and the increased risk of acute ischemic stroke may be only weakly attributable to systemic inflammation. Consequently, targeting RC alone may be ineffective in reducing inflammation-related cardiovascular events. These results highlight the need for differentiated therapeutic strategies, indicating that RC and inflammation should be addressed independently, as their contributions to cardiovascular and cerebrovascular risk appear to operate through distinct biological mechanisms.

Our study possesses several notable strengths, which we will outline below. Firstly, our study benefits from a comparatively large sample size, enhancing the statistical power and generalizability of our findings. Secondly, this study represents the first investigation to establish the independent and joint associations of RC and CHG with HT and poor prognosis following thrombolysis, thus contributing novel insights to the field. Thirdly, our study applied comprehensive statistical analyses, including subgroup analyses with interaction tests, non-linear dose–response assessments, and receiver operating characteristic curve analyses. Compared with previous studies that mainly relied on conventional regression models, these approaches allowed a more comprehensive evaluation of the robustness, heterogeneity, and predictive utility of RC and CHG. Fourth, mediation analysis revealed a significant mediating role of the SII index.

Limitations should be acknowledged. First, the retrospective design precludes causal inference and may introduce residual confounding. Second, although we accounted for a wide range of potential confounders in the multivariable models, residual confounding due to unmeasured or inadequately measured factors, such as genetic susceptibility, dietary patterns, lifestyle habits, or socioeconomic status, may still have influenced the observed associations. Third, RC was calculated indirectly from lipid profiles rather than directly measured; however, prior validation studies demonstrate high concordance between calculated and directly measured values, supporting the reliability of this approach (48). Fourth, as the study was conducted at two centers in China, generalizability to other populations may be limited. Finally, all patients received alteplase; whether these findings extend to patients treated with tenecteplase—which is increasingly used worldwide—remains to be investigated. Future research should explore the potential biological mechanisms underlying the lipid paradox in acute ischemic stroke and clarify the precise role of inflammation in this process, which may help to refine targeted therapeutic strategies.

## Conclusion

Lower RC and CHG levels at admission were associated with a higher risk of HT and poor prognosis following intravenous thrombolysis in AIS. Simultaneous assessment of RC and CHG may provide additional value in early risk stratification. Moreover, mediation analyses indicated that systemic inflammation negatively mediated these associations. These findings highlight the need to consider glycolipid metabolism and systemic inflammation as distinct therapeutic targets in the clinical management of AIS patients undergoing intravenous thrombolysis, which may help optimize individualized treatment strategies and improve clinical outcomes.

## Data Availability

The raw data supporting the conclusions of this article will be made available by the authors, without undue reservation.
