# Supplementary material for: Association of remnant cholesterol and cholesterol, high-density lipoprotein, and glucose index with unfavorable outcomes after intravenous thrombolysis in acute ischemic stroke: a dual-center cohort study
Source: Front Immunol. 2026 Mar 31;17:1797414. doi: 10.3389/fimmu.2026.1797414 (PMC13078255; doi:10.3389/fimmu.2026.1797414)
Supplement: Supplementary file 1 [file DataSheet1.pdf]

Association of remnant cholesterol and cholesterol, high-density lipoprotein, and glucose index with unfavorable outcomes after intravenous thrombolysis in acute ischemic stroke: a dual-center cohort study

Supplementary Materials:

Table S1. Analysis of threshold effects for HT.

	Adjusted OR (95% CI)	*P*-value
**CHG**		
Fitting by linear regression model	0.44 (0.32-0.60)	<0.001
Fitting by two-piecewise linear regression model		
Inflection point	2.76	
< 2.76	0.30 (0.19- 0.45)	<0.001
⩾2.76	1.16 (1.53-9.98)	0.004
Log-likelihood ratio		0.005

Threshold effect analyses: all models were adjusted for Model 3 covariates. Abbreviations: CI: confidence interval; OR: odds ratio; HT: hemorrhagic transformation; RC: remnant cholesterol; CHG: Cholesterol-High-Density Lipoprotein Glucose index;

Table S2. Analysis of threshold effects for poor prognosis.

	Adjusted OR (95% CI)	*P*-value
**RC**		
Fitting by linear regression model	0.39 (0.31-0.48)	<0.001
Fitting by two-piecewise linear regression model		
Inflection point	1.32	
< 1.32	0.29 (0.23-0.37)	<0.001
⩾1.32	1.98 (3.23-13.49)	<0.001
Log-likelihood ratio		<0.001
**CHG**		
Fitting by linear regression model	0.55 (0.44-0.69)	<0.001
Fitting by two-piecewise linear regression model		
Inflection point	2.92	
< 2.92	0.45 (0.33- 0.60)	<0.001
⩾2.92	1.17 (1.16-5.79)	0.02
Log-likelihood ratio		0.021

Threshold effect analyses: all models were adjusted for Model 3 covariates. Abbreviations: CI: confidence interval; OR: odds ratio; RC: remnant cholesterol; CHG: Cholesterol-High-Density Lipoprotein Glucose index;

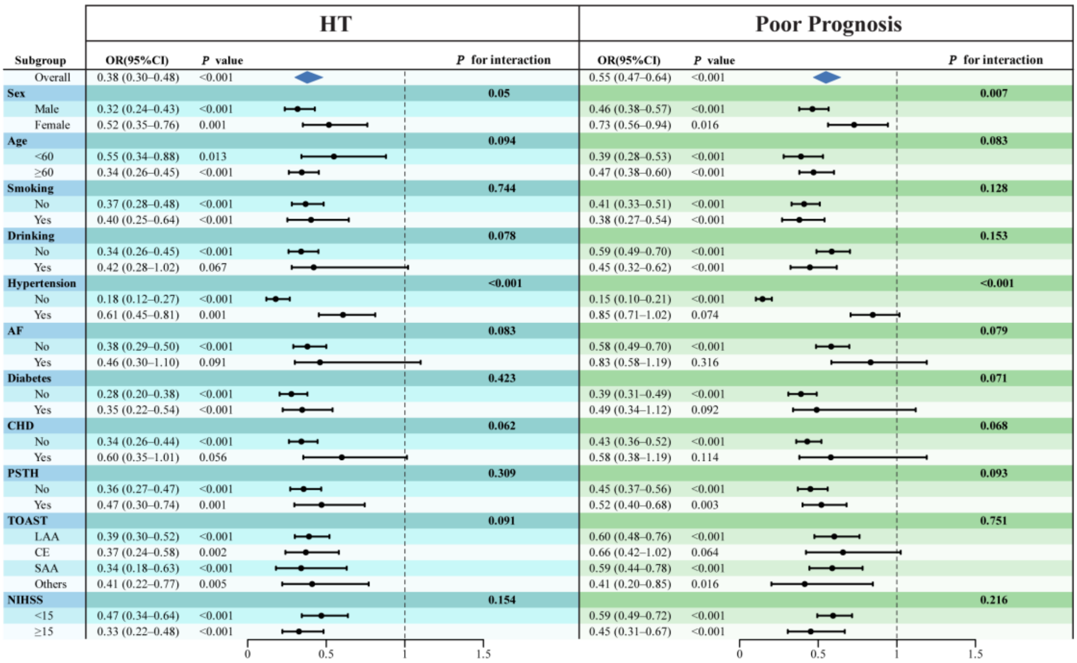

Figure S1: Subgroup analyses examining the relationship between CHG and HT, poor prognosis in AIS, adjusted for Model 3 covariates. Abbreviations: HT: hemorrhagic transformation; AF: atrial fibrillation; CHD: Coronary heart disease; PSTH: History of prior stroke or transient ischemic attack; NIHSS: National Institutes of Health Stroke Scale; TOAST: Trial of Org 10,172 in Acute Stroke Treatment; LAA: large-artery atherosclerosis; CE: cardioembolism; SAA: Small-artery occlusion; RC: remnant cholesterol; CHG: Cholesterol–High-Density Lipoprotein–Glucose index; CI: confidence interval.

Table S3. Stratified analyses of the joint association between RC and CHG with HT.

Variables	low RC & low CHG	low RC & high CHG	high RC & low CHG	high RC & high CHG	*P* for interaction
Sex					
Male	1.00 (ref)	0.60 (0.44-0.82)	0.55 (0.39-0.77)	0.18 (0.12-0.27)	0.056
Female	1.00 (ref)	1.20 (0.85-1.69)	0.95 (0.63-1.43)	0.14 (0.08-0.24)	
Age					
< 60 years	1.00 (ref)	0.95 (0.60-1.50)	0.30 (0.15-0.60)	0.08 (0.04-0.16)	0.062
≥ 60 years	1.00 (ref)	0.80 (0.60-1.07)	0.85 (0.64-1.12)	0.20 (0.14-0.29)	
Smoking					
No	1.00 (ref)	0.82 (0.62-1.09)	0.70 (0.51-0.96)	0.12 (0.08-0.18)	0.108
Yes	1.00 (ref)	0.75 (0.45-1.24)	0.68 (0.42-1.09)	0.20 (0.12-0.34)	
Drinking					
No	1.00 (ref)	0.76 (0.56-1.03)	0.70 (0.52-0.95)	0.11 (0.07-0.16)	0.067
Yes	1.00 (ref)	0.95 (0.62-1.45)	0.60 (0.36-1.00)	0.25 (0.15-0.42)	
Hypertension					
No	1.00 (ref)	0.69 (0.45-1.06)	0.70 (0.49-1.02)	0.11 (0.06-0.18)	0.162
Yes	1.00 (ref)	0.97 (0.72-1.32)	0.59 (0.40-0.85)	0.18 (0.12-0.27)	
AF					
No	1.00 (ref)	0.75 (0.55-1.03)	0.52 (0.36-0.74)	0.18 (0.12-0.26)	0.059
Yes	1.00 (ref)	1.40 (0.90-2.20)	1.10 (0.72-1.68)	0.24 (0.13-0.44)	
Diabetes					
No	1.00 (ref)	0.75 (0.53-1.06)	0.65 (0.49-0.86)	0.09 (0.06-0.15)	0.276
Yes	1.00 (ref)	0.63 (0.40-0.99)	0.64 (0.33-1.23)	0.16 (0.10-0.27)	
CHD					
No	1.00 (ref)	0.80 (0.60-1.06)	0.62 (0.47-0.82)	0.13 (0.09-0.19)	0.073
Yes	1.00 (ref)	0.85 (0.47-1.54)	0.95 (0.52-0.73)	0.22 (0.11-0.44)	
PSTH					
No	1.00 (ref)	0.72 (0.53-0.98)	0.73 (0.54-0.98)	0.13 (0.09-0.19)	0.096
Yes	1.00 (ref)	1.20 (0.76-1.88)	0.56 (0.32-0.98)	0.20 (0.10-0.37)	
TOAST					
LAA	1.00 (ref)	0.88 (0.65-1.19)	0.52 (0.35-0.78)	0.20 (0.13-0.30)	
CE	1.00 (ref)	0.90 (0.46-1.75)	0.70 (0.43-1.14)	0.35 (0.19-0.65)	0.083
SAA	1.00 (ref)	1.05 (0.50-2.22)	1.10 (0.52-2.33)	0.12 (0.02-0.83)	

Others	1.00 (ref)	0.50 (0.15-1.67)	0.70 (0.30-1.65)	0.12 (0.02-0.60)	
NIHSS					
< 15	1.00 (ref)	0.95 (0.70-1.29)	0.72 (0.52-0.99)	0.13 (0.08-0.21)	0.074
≥ 15	1.00 (ref)	0.64 (0.40-1.03)	0.75 (0.48-1.17)	0.20 (0.13-0.32)	

Patients were stratified into four groups according to the median values of RC (0.58 mmol/L) and CHG (2.58 mmol/L): group 1 (low RC & low CHG, reference), group 2 (low RC & high CHG), group 3 (high RC & low CHG), and group 4 (high RC & high CHG). Odds ratios (ORs) with 95% confidence intervals (CIs) were calculated after adjustment for Model 3 covariates. Abbreviations: HT: hemorrhagic transformation; AF: atrial fibrillation; CHD: Coronary heart disease; PSTH: History of prior stroke or transient ischemic attack; NIHSS: National Institutes of Health Stroke Scale; TOAST: Trial of Org 10,172 in Acute Stroke Treatment; LAA: large-artery atherosclerosis; CE: cardioembolism; SAA: Small-artery occlusion; RC: remnant cholesterol; CHG: Cholesterol-High-Density Lipoprotein Glucose index; CI: confidence interval.

Table S4. Stratified analyses of the joint association between RC and CHG with poor prognosis.

Variables	low RC & low CHG	low RC & high CHG	high RC & low CHG	high RC & high CHG	*P* for interaction
Sex					
Male	1.00 (ref)	0.82 (0.64-1.04)	0.76 (0.59-0.98)	0.40 (0.33-0.49)	0.068
Female	1.00 (ref)	1.49 (1.14-1.95)	0.90 (0.68-1.20)	0.38 (0.29-0.50)	
Age					
< 60 years	1.00 (ref)	0.69 (0.45-1.05)	0.61 (0.39-0.95)	0.32 (0.23-0.45)	0.121
≥ 60 years	1.00 (ref)	1.17 (0.95-1.44)	0.87 (0.70-1.07)	0.43 (0.36-0.52)	
Smoking					
No	1.00 (ref)	1.20 (0.98-1.48)	0.85 (0.67-1.07)	0.40 (0.33-0.48)	0.086
Yes	1.00 (ref)	0.55 (0.35-0.86)	0.80 (0.56-1.14)	0.42 (0.29-0.61)	
Drinking					
No	1.00 (ref)	1.03 (0.83-1.28)	0.70 (0.56-0.88)	0.38 (0.31-0.46)	0.085
Yes	1.00 (ref)	0.99 (0.68-1.44)	1.13 (0.80-1.60)	0.36 (0.25-0.51)	
Hypertension					
No	1.00 (ref)	0.58 (0.41-0.82)	0.95 (0.70-1.28)	0.13 (0.09-0.19)	0.072
Yes	1.00 (ref)	1.20 (0.98-1.47)	0.82 (0.63-1.06)	0.59 (0.48-0.72)	
AF					
No	1.00 (ref)	0.88 (0.70-1.10)	0.70 (0.54-0.90)	0.46 (0.38-0.55)	0.059
Yes	1.00 (ref)	2.10 (1.30-3.40)	1.25 (0.88-1.78)	0.28 (0.18-0.44)	
Diabetes					
No	1.00 (ref)	1.02 (0.80-1.30)	0.88 (0.71-1.08)	0.30 (0.24-0.37)	0.092

Yes	1.00 (ref)	1.15 (0.85-1.55)	0.60 (0.33-1.09)	0.72 (0.50-1.05)	
CHD					
No	1.00 (ref)	0.92 (0.73-1.16)	0.86 (0.70-1.06)	0.38 (0.32-0.45)	0.077
Yes	1.00 (ref)	1.10 (0.78-1.55)	0.58 (0.34-0.96)	0.53 (0.36-0.78)	
PSTH					
No	1.00 (ref)	0.95 (0.74-1.22)	0.70 (0.55-0.90)	0.31 (0.25-0.39)	0.081
Yes	1.00 (ref)	1.05 (0.82-1.35)	0.95 (0.70-1.29)	0.62 (0.47-0.82)	
TOAST					
LAA	1.00 (ref)	0.92 (0.70-1.20)	0.82 (0.61-1.09)	0.50 (0.39-0.64)	0.076
CE	1.00 (ref)	1.60 (1.00-2.56)	0.95 (0.62-1.45)	0.30 (0.18-0.52)	
SAA	1.00 (ref)	1.05 (0.78-1.42)	0.55 (0.37-0.81)	0.38 (0.28-0.52)	
Others	1.00 (ref)	0.50 (0.20-1.25)	1.50 (0.85-2.65)	0.60 (0.34-1.06)	
NIHSS					
< 15	1.00 (ref)	1.02 (0.83-1.27)	0.80 (0.64-1.00)	0.44 (0.36-0.53)	0.088
≥ 15	1.00 (ref)	1.08 (0.72-1.62)	1.45 (0.82-2.56)	0.36 (0.23-0.54)	

Patients were stratified into four groups according to the median values of RC (0.58 mmol/L) and CHG (2.58 mmol/L): group 1 (low RC & low CHG, reference), group 2 (low RC & high CHG), group 3 (high RC & low CHG), and group 4 (high RC & high CHG). Odds ratios (ORs) with 95% confidence intervals (CIs) were calculated after adjustment for Model 3 covariates. Abbreviations: HT: hemorrhagic transformation; AF: atrial fibrillation; CHD: Coronary heart disease; PSTH: History of prior stroke or transient ischemic attack; NIHSS: National Institutes of Health Stroke Scale; TOAST: Trial of Org 10,172 in Acute Stroke Treatment; LAA: large-artery atherosclerosis; CE: cardioembolism; SAA: Small-artery occlusion; RC: remnant cholesterol; CHG: Cholesterol-High-Density Lipoprotein Glucose index; CI: confidence interval.

Table S5. Diagnostic performance of RC, CHG, and their combination for predicting HT after IVT.

	AUC (95%CI)	*P*-Value	Optimal cut-off	Sensitivity	Specificity
**HT**					
RC	0.731 (0.707-0.755)	<0.001	0.42	0.74	0.69
CHG	0.726 (0.701-0.752)	<0.001	2.39	0.74	0.65
Combined	0.750 (0.727-0.773)	<0.001	0.82	0.79	0.60

Cutoff values were determined according to the maximum Youden index. Sensitivity and specificity values are presented for each biomarker and the combined model. Abbreviations: HT: hemorrhagic transformation; IVT: intravenous thrombolysis; ROC: receiver operating characteristic; AUC: area under the curve; CI: confidence interval; OR: odds ratio; RC: remnant cholesterol; CHG: Cholesterol-High-Density Lipoprotein Glucose index;

Table S6. Diagnostic performance of RC, CHG, and their combination for predicting poor prognosis after IVT.

	AUC (95%CI)	*P*-Value	Optimal cut-off	Sensitivity	Specificity
**Poor prognosis**					
RC	0.710 (0.693-0.727)	<0.001	0.49	0.73	0.61
CHG	0.687(0.671-0.704)	<0.001	2.76	0.48	0.80
Combined	0.721(0.705-0.737)	<0.001	0.71	0.60	0.76

Cutoff values were determined according to the maximum Youden index. Sensitivity and specificity values are presented for each biomarker and the combined model. Abbreviations: HT: hemorrhagic transformation; IVT: intravenous thrombolysis; ROC: receiver operating characteristic; AUC: area under the curve; CI: confidence interval; OR: odds ratio; RC: remnant cholesterol; CHG: Cholesterol-High-Density Lipoprotein Glucose index;

Table S7. Incremental predictive value of RC, CHG, and their combination beyond the basic model for HT and poor prognosis

**Models**	NRI (95% CI)	*P* Value	IDI (95% CI)	*P* Value
		**HT**		
Basic model	Ref		Ref	
Basic model+RC	0.550(0.480, 0.620)	<0.001	0.050(0.041, 0.059)	<0.001
Basic model+CHG	0.580(0.502, 0.660)	<0.001	0.085(0.072, 0.100)	<0.001
Basic model+RC + CHG	0.610(0.532, 0.690)	<0.001	0.097(0.082, 0.113)	<0.001
		**Poor prognosis**		
Basic model	Ref		Ref	
Basic model+RC	0.250(0.190, 0.310)	<0.001	0.016(0.012, 0.021)	<0.001
Basic model+CHG	0.300(0.240, 0.360)	<0.001	0.038(0.032, 0.045)	<0.001
Basic model+RC + CHG	0.330(0.270, 0.390)	<0.001	0.042(0.035, 0.048)	<0.001

Abbreviations: NRI: net reclassification index; IDI: integrated discrimination improvement; CI: confidence interval; HT: hemorrhagic transformation; RC: remnant cholesterol; CHG: Cholesterol-High-Density Lipoprotein Glucose index; Basic model included age, sex, smoking, drinking, hypertension, diabetes, atrial fibrillation, prior stroke or TIA, infarct distribution, baseline NIHSS score, SBP, ONT, antiplatelet therapy, anticoagulant therapy, statin use, HbA1c, and TOAST classification.
